# Low-Grade Albuminuria Is Associated with Metabolic Syndrome and Its Components in Middle-Aged and Elderly Chinese Population

**DOI:** 10.1371/journal.pone.0065597

**Published:** 2013-06-21

**Authors:** Jie Zhang, Yuhong Chen, Yu Xu, Mian Li, Tiange Wang, Baihui Xu, Jichao Sun, Min Xu, Jieli Lu, Yufang Bi

**Affiliations:** 1 Shanghai Clinical Center for Endocrine and Metabolic Diseases, Shanghai Institute of Endocrine and Metabolic Diseases, Department of Endocrine and Metabolic Diseases, Rui-jin Hospital, Shanghai Jiao-Tong University School of Medicine, Shanghai, China; 2 Key Laboratory for Endocrine and Metabolic Diseases of Ministry of Health, Rui-Jin Hospital, Shanghai Jiao-Tong University School of Medicine, E-Institute of Shanghai Universities, Shanghai, China; 3 Institute of Health Sciences, Shanghai Institutes for Biological Sciences, Chinese Academy of Sciences and Shanghai Jiao Tong University School of Medicine, Shanghai, China; RIKEN Center for Integrated Medical Science, Japan

## Abstract

**Background:**

Micro-albuminuria has been well established as one of the risk factors of metabolic syndrome (MetS). However, the association of MetS and its components with low-grade albuminuria among those with normal urinary albumin excretion has not been clearly elucidated in Chinese population.

**Methodology and Findings:**

A cross-sectional study was conducted among 9,579 participants with normal urinary albumin excretion, who were recruited from Jia Ding District, Shanghai, China. The single-void first morning urine sample was collected for urinary albumin and creatinine measurements, and urinary albumin-to-creatinine ratio (UACR) was calculated as urinary albumin divided by creatinine. Low-grade albuminuria was classified as sex-specific upper UACR quartile in this population. MetS was defined according to the National Cholesterol Education Program Adult Treatment Panel III criteria. The prevalence of MetS and its components increased across the UACR quartiles (all *P*_trend_ <0.01). A multivariable adjusted logistic regression analysis revealed that the prevalence of MetS was gradually elevated according to the UACR quartiles (adjusted odds ratios [ORs] were 1.14, 1.24 and 1.59 for UACR quartiles 2, 3 and 4, compared with the lowest quartile; *P*_trend_<0.0001). In the further stratified logistic regression analyses, the associations between low-grade albuminuria and MetS were significant in both sex strata (male and female), both age strata (<60 and ≥60 years), both body mass index strata (<24 and ≥24 kg/m^2^), and both diabetes strata (yes and no). Compared to the lowest UACR quartile, the participants in the highest quartile of UACR had the highest prevalence of central obesity (OR = 1.43; 95%CI = 1.25–1.63), high blood pressure (OR = 1.64; 95%CI = 1.43–1.87), hyperglycemia (OR = 1.52; 95%CI = 1.30–1.78) and high triglycerides (OR = 1.19; 95%CI = 1.04–1.37).

**Conclusions and Significance:**

Low-grade albuminuria was significantly associated with the increasing prevalence of MetS and its components in the middle-aged and elderly Chinese population with normal urinary albumin excretion.

## Introduction

Micro-albuminuria, defined as a urinary albumin-to-creatinine ratio (UACR) of 30–300 mg/g, was originally used to predict chronic kidney disease and diabetic nephropathy [1]. Additionally, it has been well established as one of the risk factors of cardiovascular disease (CVD) [2]–[5]. Recently, several studies have declared that the average UACR level was actually much lower, and even tiny increment of albuminuria within the previously defined normal range (low-grade albuminuria) was also associated with an increasing risk of CVD [6]–[7].

Metabolic syndrome (MetS), a cluster of metabolic disorders including central obesity, high blood pressure (BP), hyperglycemia, low high-density lipoprotein cholesterol (HDL-C) and high triglycerides, is an independent risk factor of CVD [8]. There have been several studies investigating the association between micro-albuminuria and MetS [9]–[12]. And recently, several studies have reported that there is a positive relation of albuminuria with prevalence of MetS and its related traits in non-diabetic hypertensive individuals [13], in patients with type 2 diabetes [14] and in women with polycystic ovary syndrome [15] with normoalbuminuria. However, the association between MetS and low-grade albuminuria, which also represents a risk factor of CVD, has not been well elucidated in Chinese population yet. Thus, the present study aimed to investigate the association of low-grade albuminuria with MetS and its components in a general population with normal urinary albumin excretion, for the purpose of validating the already reported findings in middle-aged and elderly Chinese population.

## Methods

### Ethics Statement

The study protocol was approved by the Institutional Review Board of the Rui-jin Hospital affiliated to Shanghai Jiao-Tong University School of Medicine. Written informed consent was obtained from each participant before data collection.

### Study Design and Participants

A total of 10,375 residents aged 40 years or older were randomly recruited from Jia Ding District, Shanghai, China between March and August 2010. The study population, design, and protocols have been described previously [16]–[18]. There were 10,337 participants who had complete information on MetS status and UACR. After further excluding participants who had a UACR level exceeding the upper limit of normal range (UACR≥30 mg/g, n = 698), or who had impaired renal function (estimated glomerular filtration rate [eGFR]<60 mL/min/1.73 m^2^ or serum creatinine [SCR]>133 µmol/L, n = 18), or who reported a history of known significant renal disease such as glomerulonephritis, nephrotic syndrome, lupus nephritis, gouty nephropathy, malignancy (n = 42), 9,579 participants (3,708 men and 5,871 women) remained in the final analytic sample.

### Clinical and Anthropometric Information

A standard questionnaire was used to obtain the information about demographic characteristics, lifestyle, history of chronic diseases and medication use with face-to-face interview by the trained investigators. The current smoker or drinker was defined as who smoked cigarettes or consumed alcohol regularly in the past 6 months. Physical activity at leisure time was estimated using the short form of the International Physical Activity Questionnaire (IPAQ) [19] by asking questions on duration of mild/moderate/vigorous activities every day and total metabolic equivalent (MET)-minutes/week was calculated for each person. High level of leisure-time physical activity was defined according to the highest tertile of total MET-minutes/week. The current uses of antihypertensive and hypoglycemic medications were recorded (yes/no) and the type of hypoglycemic drug was also collected (insulin/oral medication/Chinese herbal medication/others) for each self-reported diabetic participant.

Body height and weight were measured to the nearest 0.1 cm and 0.1 kg, respectively, while participants were wearing light clothes and no shoes. Body mass index (BMI) was calculated as body weight in kilograms divided by body height squared in meters (kg/m^2^). Waist circumference was measured to the nearest 0.1 cm at umbilical level in a standing position. BP was measured with an automated electronic device (OMRON Model HEM-752 FUZZY, Omron Company, Dalian, China) at a non-dominant arm 3 times consecutively with 1-minute interval each after at least 5 minutes rest in a seated position. The three readings of systolic BP (SBP) and diastolic BP (DBP) were averaged for analysis.

### Laboratory Measurements

A single-void first morning urine sample was collected. Urinary albumin and creatinine were measured using immunoturbidimetric method (Beijing Atom High-Tech, Beijing, China) and Jaffe’s kinetic method on an automatic analyzer (Hitachi 7600-020, Tokyo, Japan), respectively. The UACR in mg/g was calculated as urinary albumin concentration divided by creatinine concentration. All participants underwent a 75-g oral glucose tolerance test (OGTT) after an overnight fast of more than 10 hours. Fasting plasma glucose (FPG), OGTT 2-hour plasma glucose (OGTT-2 h PG), triglycerides, total cholesterol (TC), low-density lipoprotein cholesterol (LDL-C), HDL-C, aminotransferase (ALT), aspartate aminotransferase (AST), and γ-glutamyltransferase (GGT), albumin (ALB), SCR were determined using an autoanalyser (ADVIA-1650 Chemistry System, Bayer corporation, Germany). The abbreviated Modification of Diet in Renal Disease (MDRD) formula recalibrated for Chinese was used to calculate eGFR expressed in mL/min/1.73 m^2^: eGFR = 186 × [SCR × 0.011] ^−1.154^ × [age] ^−0.203^ × [0.742 if female] × 1.233, where SCR is expressed as µmol/L and 1.233 is the adjusting coefficient for Chinese [20]. Serum fasting insulin (FINS) concentrations were measured by an electrochemiluminescence assay (Roche-Diagnositics, Switherland). The index of homeostasis model assessment of insulin resistance (HOMA-IR) was calculated according to the formula: HOMA-IR = FINS concentrations (c) × FPG concentrations (mmol/L)/22.5.

### Definition

The MetS was defined according to the National Cholesterol Education Program Adult Treatment Panel III (NCEP-ATP III) criteria [21] with modification on waist circumference cutoff for Asian population suggested by the 1998 World Health Organization Asian Pacific Guideline [22]. The MetS was defined as having three or more of the following factors: (1) Central obesity: waist circumference ≥90 cm in men and ≥80 cm in women; (2) High BP: BP≥130/85 mmHg or taking antihypertensive medication; (3) High triglycerides: triglycerides≥1.69 mmol/L (150 mg/dL); (4) Low HDL-C: HDL-C<1.03 mmol/L (40 mg/dL) in men and <1.29 mmol/L (50 mg/dL) in women; (5) Hyperglycemia: FPG≥6.1 mmol/L (110 mg/dL) or taking antidiabetic medication.

The sex-specific cut-off points of UACR quartiles were as follows: quartile 1 (Q1): 0–2.27 (men) and 0–3.08 (women) mg/g; quartile 2 (Q2): 2.28–3.66 (men) and 3.09–5.21 (women) mg/g; quartile 3 (Q3): 3.67–6.20 (men) and 5.22–8.80 (women) mg/g; quartile 4 (Q4): 6.21–29.9 (men) and 8.81–29.9 (women) mg/g. In the present study, participants in the sex-specific upper quartile of UACR were classified as having low-grade albuminuria.

### Statistical Analysis

All data were analyzed using SAS version 8.2 (SAS Institute Inc, Cary, NC, USA). Continuous variables were presented as means (±SD) or medians (interquartile ranges) for skewed variables. Categorical variables were shown in proportions. Triglycerides, HOMA-IR, FINS, ALT, AST, GGT and UACR were logarithmically transformed before analysis because of the skewed distributions.

The study participants were divided into four groups according to sex-specific quartiles of UACR. Anthropometric and laboratory features in each quartile were described, and *P*_trend_ across quartiles was tested using linear regression analysis and Cochran-Mantel-Haenszel (CMH) for continuous and categorical variables, respectively. In order to investigate the association between UACR and metabolic risk factors, simple regression and multiple stepwise linear regression analyses were used.

The odds ratios (ORs) of having MetS for different quartiles of UACR were calculated using logistic regression analyses. Models were adjusted for sex (male/female), age, BMI, LDL-C, FINS, eGFR, ALB, current smoking status (yes/no), and family history of diabetes (yes/no). To further investigate whether UACR was an independent risk factor for the prevalence of MetS in different subgroups, a stratified analysis was further conducted by the major risk factors including sex (male and female), age (<60 and ≥60 years, representing middle-aged or elderly population), BMI (<24 and ≥24 kg/m^2^, representing normal weight or overweight/obese in Chinese population) [23]), diabetes (yes and no), and hypertension (yes and no). The ORs of the prevalence of MetS components for UACR were also adjusted for sex (male/female), age, BMI, LDL-C, FINS, eGFR, ALB, current smoking (yes/no), and family history of diabetes (yes/no) (Model 1) and additionally adjusted for other components of MetS as dichotomized variables on the basis of Model 1 (Model 2). Tests of linear trend across increasing quartiles of UACR were conducted by treating the quartiles as a continuous variable. Results were presented as ORs and 95% confidence interval (CI). Significance tests were two-tailed and a *P*<0.05 was stated as statistically significant.

## Results

### Characteristics of the Study Population

Among the 9,579 participants, the prevalence of MetS was 36.5% and it was lower in male than in female (30.7 vs. 40.2%, *P*<0.0001). Similarly, the median of UACR in male was significantly lower than that in female (3.67 vs. 5.22 mg/g, *P*<0.0001). Compared to those without MetS, participants with MetS were more likely to be female, elderly person, and had significantly higher levels of BMI, waist circumference, SBP and DBP, FPG, OGTT-2 h PG, TC, triglycerides, LDL-C and lower levels of HDL-C (all *P*<0.01). However, those with MetS were more frequent in absence of current smoking or drinking status and more active in taking part in vigorous physical activities during their leisure time (Table 1). There also was a statistically significance between participants with and those without MetS in UACR (5.29 vs. 4.21 mg/g, *P*<0.0001).

**Table 1:** General characteristics of the study population.

	MetS Status		UACR quartiles				
	Non MetS	MetS	Q1	Q2	Q3	Q4	*P*_trend_
	(n = 6083)	(n = 3496)	(n = 2396)	(n = 2394)	(n = 2399)	(n = 2390)	
UACR (mg/g)	4.21(2.53–7.07)	5.29(3.06–9.32) *	1.86(1.39–2.28)	3.58(3.13–4.34)	5.89(5.02–7.00)	12.29(9.48–22.37)	-
Male, n(%)	2571(42.3)	1137(32.5) *	929(38.8)	923(38.6)	932(38.9)	924(38.7)	0.9428
Age (years)	57.7±9.8	59.4±9.1*	57.4±9.3	57.2±89.2	58.4±9.4	60.2±9.9	<0.0001
Current smoking, n(%)	1391(22.9)	593(17.0) *	457(19.1)	500(20.9)	538(22.4)	489(20.5)	0.1231
Current drinking, n(%)	657(10.8)	299(8.6) #	231(9.6)	235(9.8)	236(9.8)	254(10.6)	0.2769
High level of leisure-time physical activity, n(%)	2838(46.7)	1821(52.1) *	1166(48.7)	1138(47.5)	1191(49.7)	1164(48.7)	0.6258
BMI (kg/m^2^)	24.0±2.89	26.9±2.9 *	24.8±3.2	24.8±3.2	25.1±3.1	25.6±3.4	<0.0001
Waist circumference (cm)	79.4±8.1	87.9±7.6 *	81.7±8.7	81.8±8.7	82.6±8.7	83.9±9.4	<0.0001
SBP (mmHg)	135.9±19.3	147.9±17.8	137.4±19.1	137.4±19.5	140.4±19.0	145.8±19.9	<0.0001
DBP (mmHg)	80.7±9.9	85.7±9.8 *	82.0±9.8	81.5±10.1	82.7±10.1	84.0±10.6	<0.0001
Triglycerides (mmol/L)	1.12(0.85–1.46)	2.03(1.58–2.74) *	1.33(0.96–1.85)	1.36(0.95–1.91)	1.36(0.99–1.94)	1.43(0.99–2.07)	<0.0001
TC (mmol/L)	5.25±0.94	5.47±1.11 *	5.36±0.99	5.30±0.98	5.30±1.01	5.37±1.04	0.7076
HDL-C (mmol/L)	1.43±0.31	1.14±0.25 *	1.35±0.31	1.33±0.31	1.32±0.32	1.31±0.33	<0.0001
LDL-C (mmol/L)	3.13±0.81	3.29±0.92 *	3.21±0.85	3.19±0.85	3.15±0.84	3.20±0.89	0.5810
FPG (mmol/L)	5.18±0.97	6.05±1.82 *	5.38±1.15	5.34±1.19	5.46±1.24	5.81±1.88	<0.0001
OGTT-2 h PG (mmol/L)	7.03±3.04	9.99±5.00 *	7.67±3.60	7.73±3.71	8.00±3.87	9.05±5.03	<0.0001
HOMA-IR	1.28(0.88–1.82)	2.42(1.67–3.53) *	1.56(1.10–2.32)	1.49(1.00–2.27)	1.57(0.99–2.45)	1.78(1.13–2.76)	0.0336
FINS (mIU/L)	5.70(4.00–7.90)	9.50(6.80–13.00) *	6.80(4.90–9.46)	6.52(4.40–9.50)	6.73(4.40–9.90)	7.30(4.80–10.80)	0.0035
ALT (U/L)	16.8(13.0–22.6)	21.0(15.5–29.7) *	17.6(13.3–24.2)	17.7(13.5–24.3)	18.0(13.7–25.6)	18.7(14.5–26.7)	<0.0001
AST (U/L)	21.2(18.1–25.0)	21.8(18.6–26.4) *	21.2(18.3–25.1)	21.2(18.1–24.9)	21.5(18.2–25.8)	22.0(18.5–26.2)	<0.0001
GGT (U/L)	19.0(14.0–29.0)	26.0(19.0–42.0) *	20.0(14.0–31.0)	21.0(14.0–32.0)	22.0(15.0–35.0)	24.0(16.0–39.0)	<0.0001
ALB (g/L)	48.6±2.4	48.9±2.3 *	48.8±2.4	48.7±2.3	48.8±2.3	48.8±2.5	0.3212
eGFR (mL/min/1.73 m^2^)	134.1±23.8	131.9±25.9*	129.2±23.0	132.2±22.5	135.4±24.9	136.3±27.3	<0.0001

Data were means±SD or medians (interquartile range) for skewed variables, or proportions for categorical variables;

* *P*<0.0001 comparing to Non MetS group; #: *P*<0.001 comparing to Non MetS group;

*P*_trend_ was calculated from CMH chi-square tests for categorical variables and linear regression analysis for continuous variables.

Abbreviations: MetS, metabolic syndrome; UACR, urinary albumin-to-creatinine ratio; BMI, body mass index; SBP, systolic blood pressure; DBP, diastolic blood pressure; TC, total cholesterol; HDL-C, high-density lipoprotein cholesterol; LDL-C, low-density lipoprotein; FPG, fasting plasma glucose; OGTT-2 h PG, OGTT 2-hour plasma glucose; HOMA-IR, homeostasis model assessment of insulin resistance; FINS, fasting serum insulin; ALT, aminotransferase; AST, aspartate aminotransferase; GGT, γ-glutamyltransferase; ALB, albumin; eGFR, estimate glomerular filtration rate;

Because of the statistical difference in UACR values between male and female, the study population was divided into four groups by sex-specific UACR quartiles.

(UACR quartiles cut-off points for male: 0 mg/g<Q1<2.28 mg/g, 2.28 mg/g ≤Q2<3.67 mg/g, 3.67 mg/g≤Q3<6.21 mg/g, 6.21 mg/g≤Q4<30.00 mg/g; and for female: 0 mg/g<Q1<3.09 mg/g, 3.09m g/g ≤Q2<5.22 mg/g, 5.22 mg/g≤Q3<8.81 mg/g, 8.81 mg/g≤Q4<30.00 mg/g).

Then participants were divided into four groups according to sex-specific quartiles of UACR. Clinical and biochemical characteristics of the four groups were shown in Table 1.There was no significant difference among the four groups for sex distributions, current smoking or drinking status, levels of physical activities during leisure time, and levels of TC, LDL-C and ALB. Nevertheless, other metabolic risk factors including age, BMI, waist circumference, SBP and DBP, triglycerides, FPG and OGTT-2h PG, HOMA-IR, FINS, liver enzymes and eGFR increased significantly with the increment of UACR, whereas HDL-C decreased (all *P*_trend_ <0.05).

### Association between UACR and Metabolic Risk Factors

Simple regression analyses revealed that age, sex, BMI, waist circumference, SBP and DBP, triglycerides, FPG, eGFR, and current smoking and drinking status were significantly associated with UACR (Table 2). After performing multivariate stepwise linear regression analysis, we found that UACR was positively and significantly associated with sex, eGFR, age, SBP, FPG, waist circumference and current smoking status (standardized estimate from 0.0441 to 0.2467, all *P*<0.01), while HDL-C was inversely associated with UACR (standardized estimate was −0.0321, *P* = 0.0023).

**Table 2:** Simple and multiple stepwise linear regression analysis of metabolic risk factors associated with UACR.

	Univariate Model		Multivariate Model		
	β±SE	*P* value	β±SE	Std β	*P* value
Sex (male = 1, female = 2)	0.1371±0.0070	<0.0001	0.1728±0.0562	0.2467	<0.0001
Age (years)	0.0042±0.0004	<0.0001	0.0049±0.0090	0.1385	<0.0001
BMI (kg/m^2^)	0.0087±0.0011	<0.0001	–	–	–
Waist circumference (cm)	0.0018±0.0004	<0.0001	0.0020±0.0004	0.0535	<0.0001
SBP (mmHg)	0.0029±0.0002	<0.0001	0.0019±0.0002	0.1118	<0.0001
DBP (mmHg)	0.0018±0.0003	<0.0001	–	–	–
Triglycerides (mmol/L)	0.0783±0.0149	<0.0001	–	–	–
HDL-C (mmol/L)	−0.0002±0.0110	0.9828	−0.0344±0.0113	−0.0321	0.0023
FPG (mmol/L)	0.0284±0.0025	<0.0001	0.0168±0.0024	0.0696	<0.0001
eGFR (mL/min/1.73 m^2^)	0.0016±0.0001	<0.0001	0.0022±0.0001	0.1621	<0.0001
Current smoking (yes = 1, no = 0)	−0.094±0.0086	<0.0001	0.0371±0.0106	0.0441	0.0005
Current drinking (yes = 1, no = 0)	−0.0720±0.0116	<0.0001	–	–	–
High level of leisure-time physical activity (yes = 1, no = 0)	0.0061±0.0070	0.3856	–	–	–

Abbreviations: BMI, body mass index; SBP, systolic blood pressure; DBP, diastolic blood pressure; HDL-C, high-density lipoprotein cholesterol; FPG, fasting plasma glucose; eGFR, estimate glomerular filtration rate.

### The Prevalence of MetS and its Components According to UACR Quartiles

**Figure 1:**
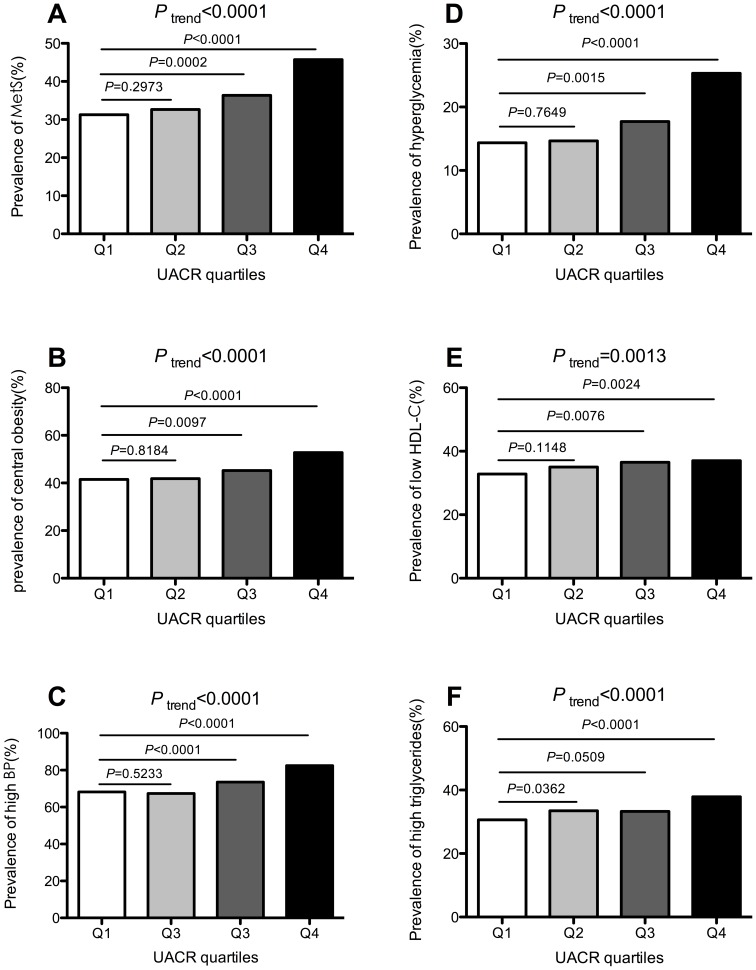
Prevalence of MetS (Panel A), central obesity (Panel B), high BP (Panel C), hyperglycemia (Panel D), low HDL-C (Panel E), high triglycerides (Panel F) in different UACR quartiles; UACR quartiles cut-off points for male: 0 mg/g<Q1<2.28 mg/g, 2.28 mg/g ≤Q2<3.67 mg/g, 3.67 mg/g≤Q3<6.21 mg/g, 6.21 mg/g≤Q4<30.00 mg/g; and for female: 0 mg/g<Q1<3.09 mg/g, 3.09 mg/g ≤Q2<5.22 mg/g, 5.22 mg/g≤Q3<8.81 mg/g, 8.81 mg/g≤Q4<30.00 mg/g); *P* values and *P*_trend_ were calculated from CMH chi-square tests. **Panel A**: The prevalence was 31.3, 32.7, 36.4 and 45.7% for MetS from the lowest to the highest UACR quartile, respectively. **Panel B**: The prevalence was 41.5, 41.9, 45.2 and 52.8% for central obesity from the lowest to the highest UACR quartile, respectively. **Panel C**: The prevalence was 68.2, 67.3, 73.5 and 82.5% for high BP from the lowest to the highest UACR quartile, respectively. **Panel D**: The prevalence was 14.4, 14.7, 17.7 and 25.3% for hyperglycemia from the lowest to the highest UACR quartile, respectively. **Panel E**: The prevalence was 32.9, 35.0, 36.5 and 37.0% for low HDL-C from the lowest to the highest UACR quartile, respectively. **Panel F**: The prevalence was 30.6, 33.5, 33.3 and 37.9% for high triglycerides from th lowest to the highest UACR quartile, respectively.

The prevalence of MetS and its components in the different UACR quartiles was shown in Figure 1. From the lowest UACR quartile across to the highest one, the prevalence of MetS was 31.3, 32.7, 36.4 and 45.7%, respectively (*P*_trend_<0.0001, Panel A). Strikingly, a significant increase was observed in the third and the highest quartiles compared to the lowest one (*P* = 0.0021 and *P<*0.0001, respectively). Similarly, the prevalence of MetS components including central obesity, high BP, hyperglycemia, low HDL-C and high triglycerides also increased across the UACR quartiles (all *P*_trend_<0.01). Compared to the lowest UACR quartile, the third and highest quartiles showed a significant increase in the prevalence of central obesity, high BP, hyperglycemia and low HDL-C (all *P*<0.01). And a significant increase was observed in the highest UACR quartile compared to lowest one in the prevalence of high triglycerides (*P*<0.0001).

### Association between Low-grade Albuminuria and Prevalence of MetS

Table 3 revealed that the prevalence of MetS was gradually elevated according to the UACR quartiles (ORs were 1.14, 1.24 and 1.59 for UACR quartiles 2, 3 and 4, compared with the lowest quartile; *P*_trend_<0.0001) in multivariate logistic analyses. Furthermore, the prevalence of MetS was also increased along with the increasing UACR quartiles in all dichotomous subgroups (all *P*_trend_<0.05). And compared to the lowest UACR quartile, participants in the highest one showed the high prevalence of MetS both in male (OR = 1.83; 95%CI = 1.50–2.32) and female (OR = 1.31; 95%CI = 1.15–1.64), middle-aged (OR = 1.42; 95%CI = 1.17–1.71) and elderly (OR = 1.75; 95%CI = 1.41–2.18) participants, normal weight (OR = 1.56; 95%CI = 1.17–2.08) and overweight/obesity (OR = 1.59; 95%CI = 1.36–1.87) participants, those who with (OR = 1.53; 95%CI = 1.07–2.17) and without (OR = 1.43; 95%CI = 1.22–1.68) diabetes and those who with hypertension (OR = 1.52; 95%CI = 1.28–1.81).

**Table 3:** Elevated UACR quartiles associated with the prevalence of MetS in total and stratified population.

		UACR quartiles OR (95%CI)				
		Q1	Q2	Q3	Q4	*P*_trend_
Total		1.00	1.14(0.99–1.32)	1.24(1.08–1.43)	1.59(1.38–1.83)	<0.0001
Sex	Male (n = 3708)	1.00	1.14(0.90–1.45)	1.35(1.06–1.71)	1.83(1.50–2.32)	<0.0001
	Female (n = 5871)	1.00	1.13(0.95–1.35)	1.15(0.97–1.38)	1.31(1.15–1.64)	0.0008
Age	<60 yr (n = 5739)	1.00	1.09(0.91–1.31)	1.20(1.00–1.44)	1.42(1.17–1.71)	0.0002
	≥60 yr (n = 3840)	1.00	1.21(0.96–1.52)	1.26(1.01–1.57)	1.75(1.41–2.18)	<0.0001
BMI	<24 kg/m^2^ (n = 3689)	1.00	1.08(0.81–1.44)	1.20(0.90–1.60)	1.56(1.17–2.08)	0.0022
	≥24 kg/m^2^ (n = 5890)	1.00	1.17(0.99–1.38)	1.25(1.06–1.47)	1.59(1.36–1.87)	<0.0001
Diabetes	Yes (n = 1617)	1.00	1.12(0.77–1.64)	1.42(0.98–2.08)	1.53(1.07–2.17)	0.0098
	No (n = 7962)	1.00	1.15(0.98–1.35)	1.20(1.03–1.41)	1.43(1.22–1.68)	<0.0001
Hypertension	Yes (n = 5552)	1.00	1.24(1.04–1.49)	1.19(0.99–1.42)	1.52(1.28–1.81)	0.0001
	No (n = 4027)	1.00	0.97(0.76–1.24)	1.24(0.97–1.59)	1.24(0.95–1.62)	0.0351

Abbreviations: OR, odds ratio; CI: confidence interval; MetS, metabolic syndrome;

Models were adjusted for sex (except for sex strata), age, BMI, LDL-C, FINS, eGFR, serum albumin, smoking status, family history of diabetes.

### Association between Low-grade Albuminuria and the Prevalence of MetS Components

UACR was independently associated with the increasing prevalence of MetS components while adjusting for some traditional cardiometabolic risk factors (all *P*_trend_<0.05, Table 4). After further adjusting for other components of MetS, the associations of UACR with individual components of MetS were still significant for the prevalence of central obesity, high BP, hyperglycemia and high triglycerides (all *P*_trend_<0.05), with the exception for low HDL-C (*P*_trend_ = 0.1194) and comparing to the lowest UACR quartile, participants in the highest one showed a higher prevalence of central obesity (OR = 1.43; 95%CI = 1.25–1.63), high BP (OR = 1.64; 95%CI = 1.43–1.87), hyperglycemia (OR = 1.52, 95%CI 1.30–1.78) and high triglycerides (OR = 1.19, 95%CI = 1.04–1.37).

**Table 4:** Elevated UACR quartiles associated with the prevalence of MetS components.

		UACR quartiles OR (95%CI)				
		Q1	Q2	Q3	Q4	*P*_trend_
Central obesity	Model 1	1.00	1.09(0.96–1.25)	1.28(1.12–1.46)	1.58(1.38–1.80)	<0.0001
	Model 2	1.00	1.07(0.94–1.22)	1.21(1.06–1.39)	1.43(1.25–1.63)	<0.0001
High BP	Model 1	1.00	1.07(0.94–1.21)	1.16(1.02–1.32)	1.71(1.50–1.95)	<0.0001
	Model 2	1.00	1.06(0.93–1.20)	1.15(1.01–1.30)	1.64(1.43–1.87)	<0.0001
Hyperglycemia	Model 1	1.00	1.04(0.88–1.23)	1.16(0.98–1.37)	1.61(1.37–1.88)	<0.0001
	Model 2	1.00	1.03(0.87–1.22)	1.13(0.96–1.33)	1.52(1.30–1.78)	<0.0001
Low HDL-C	Model 1	1.00	1.15(1.01–1.31)	1.23(1.08–1.40)	1.17(1.03–1.33)	0.0106
	Model 2	1.00	1.09(0.95–1.25)	1.19(0.99–1.36)	1.09(0.95–1.25)	0.1194
High Triglycerides	Model 1	1.00	1.24(1.09–1.41)	1.16(1.02–1.32)	1.28(1.12–1.45)	0.0017
	Model 2	1.00	1.18(1.03–1.35)	1.08(0.94–1.24)	1.19(1.04–1.37)	0.0479

Abbreviations: OR, odds ratio; CI: confidence interval; BP, blood pressure; HDL-C, high-density lipoprotein cholesterol; BMI, body mass index;

Model 1 adjusted for sex, age, BMI (except for central obesity), LDL-C, FINS, eGFR, serum albumin, smoking status, family history of diabetes;

Model 2 adjusted for all confounders in Model 1 plus central obesity (except for central obesity), high BP (except for high BP), hyperglycemia (except for hyperglycemia), low HDL (except for low HDL-C), high triglycerides (except for high triglycerides).

## Discussion

In the present study, we found that elevated UACR within the normal range of micro-albuminuria threshold (30 mg/g) was significantly associated with the increasing prevalence of MetS and its components in middle-aged and elderly Chinese population, even for non-diabetic participants. The magnitude of this association persisted after controlling for traditional cardiometabolic risk factors and other components of MetS.

Previous studies have confirmed that there is an increased risk of CVD and mortality associating with micro-albuminuria [24]–[28]. However, recent studies demonstrated that urinary albumin excretion less than the current micro-albuminuria threshold (30 mg/g) also significantly predicted CVD [2], [6]–[7], [29]–[32]. Data from the HOPE study showed that the risk of cardiovascular events increased with the UACR level and suggested that any degree of albuminuria is a risk factor for CVD [2]. Another data from the Framingham Heart Study indicated that low-grade urinary albumin excretion less than the current micro-albuminuria threshold predicted a more rapid progression toward vascular diseases in healthy subjects, and in the long term, an increased incidence of cardiovascular events [6]–[7]. The study by Arnlöv et al. reported that the threshold for an elevated cardiovascular event rate was 3.9 mg/g of UACR for men and 7.5 mg/g of UACR for women [7]. The MONICA/KORA Augsburg Echocardiographic Substudy also reported that low-grade albuminuria with UACR of 4.0–10.0 mg/g was associated with an increased cardiovascular risk [31].

Therefore, low-grade albuminuria is well established as one of the risk factors for CVD in other nations and might be associated with MetS and its components. A Korean study indicated that low-grade albuminuria in Korean men was associated with a variety of cardiovascular risk factors and MetS, and this finding was unchanged when they excluded participants with diabetes and hypertension; however, the association of low-grade albuminuria with hypertension and MetS was vanished in a multiple-adjusted model [33]. In our study, we confirmed the association between low-grade albuminuria and MetS in both genders in a general Chinese population and further adjustment for a wide spectrum of lifestyle and biochemical risk factors did not materially change the significance of this association. Moreover, compared with the latter study, our study population was much larger, which allowed a careful control for the potential confounding effects using a stratified analysis and detected significant associations between low-grade albuminuria and MetS either in male or female, in middle-aged or elderly participants, in normal weights or overweight/obesity participants, in diabetes or non-diabetes and in those who with hypertension.

The potential pathophysiological mechanism linking low-grade albuminuria to MetS and its components are not fully established and inconsistent, and further research is ongoing. A previous study have hypothesized that albuminuria could result from generalized vascular leakiness rather than a specific renal lesion [34]. The passage of albumin and other macromolecules into the vessel wall culminates in an inflammatory response, lipid accumulation, and eventually atherosclerosis. This widespread vascular disorder progresses to loss of vessel distension, increasing pressures in the kidneys [35]. Thus albuminuria has been associated with hypertension [32], [36]–[39], hyperlipidemia [32], [39], diabetes, insulin resistance [40]–[41], and inflammation [32], [42], which are the components of MetS and the risk factors for CVD. However, the causal association of the elevated urinary albumin excretion with cardiometabolic risk factors was still ambiguous. Data from the Prevention of Renal and Vascular End Stage Disease (PREVEND) study showed that urinary albumin exertion independently predicted type 2 diabetes during a mean follow-up period of 4.2 years [43]. And data from an Epidemiological Study on the Insulin Resistance syndrome (DESIR) study also demonstrated that elevated urinary albumin exertion predicted the 9-year risk of diabetes in men, independent of baseline or early development of metabolic abnormalities or insulin resistance [44]. Meanwhile, the study by Bonnet et al. showed that subjects with a high waist circumference or with MetS at baseline were more likely to develop elevated albuminuria at 6 years compared with those with a normal waist circumference or absence of MetS [45]. In our study, low-grade albuminuria was positively associated with the prevalence of MetS and its components including central obesity, high BP, hyperglycemia and high triglycerides, and the results were not changed in participants without diabetes.

Thus, like previous studies, our findings add more evidence for the hypothesis that very low degrees of urinary albumin excretion, below the conventional threshold for micro-albuminuria, may also has increased cardiometabolic risks in Chinese population. Nevertheless, there are several limitations to be mentioned. First, because of the cross-sectional nature of this study, we cannot conclude a causal association between MetS and low-grade albuminuria. We will pursue to examine the prospective association of low-grade albuminuria with MetS and cardiovascular risk in future when the data are available. Second, urinary albumin excretion was assessed in a single urine sample. We acknowledge that a 24-h urine sample or three samples from 3 consecutive days would provide more stable measures for urinary albumin excretion [26]. However, it was reported that the use of spot specimens for UACR was more convenient for a large cohort sample and agreed with 24-h urinary albumin excretion well, and could be used as a reliable alternative to 24-h urinary albumin excretion or multiple samples in large epidemiological research [46]–[47]. Third, although wide spectrum of covariates had been included in the adjustment, some residual or undetected confounding factors could not be ruled out.

In conclusion, our study demonstrated that in a middle-aged and elderly Chinese population with the UACR less than the current micro-albuminuria threshold (<30 mg/g), low-grade albuminuria was significantly associated with the increasing prevalence of MetS and its components.
